# Book Review

**Published:** 1944

**Authors:** 


### Reviews of Books

**The Medical Annual, 1944.** Edited by Sir Henry Tidy, K.B.E., and A. Rendle Short, M.D., F.R.C.S. Pp. xxvi., 404 (71). With 40 plates. Bristol: John Wright & Sons Ltd. Price 25s. net.—Readers will be glad to know that a slightly larger number of copies of the edition of 1944 has been printed owing to some concession by the Ministry of Supply. Hitherto the war editions have been far smaller than the number of would-be-purchasers. Wright's *Medical Annual* remains the favourite Compendium of the English speaking medical world. The 1944 edition is full of up-to-date and important items. Among these should be mentioned infective hepatitis or catarrhal jaundice, which is now at last adequately explained. In the article on radiology, mass radiography of the chest is critically and cautiously appraised. There is some hint that it may take its place among other Government schemes for making "worlds fit for heroes." Radiotherapy is admirably reviewed. The importance of malaria at every stage of the world war cannot be exaggerated, and there are two excellent articles—one by Manson-Bahr on the general problems of malaria, and another by Heathcote on quinine and its substitutes in the treatment of malaria. The directions for treating cases of relapse in Great Britain are inadequate, not from any fault of the reviewers but because the subject has not met with sufficient attention since the late Professor Warrington Yorke's masterly article in the *B.M.J.* of July 18, 1942. The various articles on war wounds, war surgery and burns will repay careful reading; so too will Aubrey Lewis's summary on war psychiatry. But every reader will pick his own subjects, and none is likely to be disappointed. Wright's *Medical Annual* is as good as usual.

**The History of Cæsarean Section.** By J. H. Young, M.B., Ch.B. Pp. viii., 253. London: H. K. Lewis & Co. Ltd. 1944. Price 16s. net.—This book is remarkable in that so much of interest and instruction has been packed into such a small compass: for probably every student and obstetrician, or would-be obstetrician, will find here matter of help to him in his practice. The book and its bibliography will also give great satisfaction to the historian, as he reads of the early efforts to win success in this operation, efforts which seemed to have failed until the opening of the present century. Then the latest methods of performing the operation having been proved safe, it became for a time almost fashionable and the cure-all for many obstetric difficulties. One cannot help being impressed with the many conditions which have been treated by this operation for a time, only to give place later to other more conservative treatments. As with the indication so with the methods. We are shown the mistakes in methods of doing the operation from the days when the uterus was not sutured until the present day. Similarly at various times many and varied incisions have been used in all parts of the uterus : fundus, back, cervix, lower segment, etc., and in all directions, diagonal, vertical and transverse. In fact, if anyone contemplated doing a "new" method either of incision or suturing, it would be very difficult to find one which is not described in the book as having already been "tried" and—in many instances—"found wanting."

**Synopsis of Surgical Anatomy.** Fifth Edition. A. LEE MACGREGOR, M.Ch., F.R.C.S. Bristol : John Wright & Sons Ltd. 1943. Price 25s. net.—The fact that two reprints of this book, followed by the present Fifth Edition, have been required since the outbreak of the war are a sure indication that it meets the requirements of those for whom it has been produced. The present edition shows evidence of thorough revision, and a good deal of new matter has been added, particularly in the section devoted to the autonomic nervous system. This revision has been most thoroughly done, and an enormous amount of information is contained in the pages devoted to it. The whole work is packed with anatomical detail arranged in a convenient way for ready reference. Here and there, perhaps, can be found instances where a little more detail would not be out of place, witness the rather casual reference to Meckel's diverticulum. On the whole the book is complete and accurate, and likely to be of the greatest use to those for whom it has been written. It is, perhaps, rather strong meat to be used as a systematic text-book for the student, to be read from cover to cover. As a book of reference, however, and to those reading for higher qualifications, it should meet a real want. The illustrations are numerous and helpful, and the index is adequate. A final word of congratulation should be awarded to the publishers for the way they have overcome the difficulties of book-production under present circumstances.

**Symptoms and Signs in Clinical Medicine.** Third Edition. By E. NOBLE CHAMBERLAIN, M.D., M.Sc., F.R.C.P. Pp. viii., 456 ; 346 illustrations. Bristol : John Wright & Sons Ltd. 1943. Price 30s.—There is really little to add to the whole-hearted commendation expressed when this excellent work first appeared. The book is sumptuously produced and beautifully printed. How the publishers have been able to do such work in view of their difficult experiences is in itself a problem. The book itself is somewhat expanded : a chapter on Radiology is added. We note with interest (and perhaps a little relief) that the author still believes in mitral regurgitation. It is difficult to think of a better guide for the student in his approach to clinical medicine : accurate, interesting and concise. Modern students are indeed fortunate to be provided with such an aid to intelligent examination and diagnosis.

**Electrocardiograms.** Second Edition. By H. WALLACE JONES, M.D., M.Sc., F.R.C.P., and E. NOBLE CHAMBERLAIN, M.D., M.Sc., F.R.C.P. Pp. 56. Illustrated. Bristol : John Wright & Sons Ltd. 1943. Price 5s. net.—The appearance of a second edition of this atlas in four years is sufficient proof that it has proved of value to students and practitioners. This is hardly surprising. The book is well produced with excellent illustrations. The text is clear and simple. This atlas thus provides a very good introduction to electrocardiography without going deeply into theoretical considerations which are of no practical importance to the ordinary student. Apart from illustrating the main features of the electrocardiogram it indicates the type of case in which this method of investigation is likely to be of value to practitioners. (The only error noted is that Fig. 49 has been printed upside down.) It can be thoroughly recommended.

**First Aid Treatment of Home Guard Casualties.** By THOMAS HENRY MEEK, M.D. Pp. vii., 55. London: H. K. Lewis & Co. Ltd. 1943. Price 4s. net.—This booklet attempts to explain to the Home Guard the essentials of first-aid treatment in the field. The material, presented concisely in non-technical language, is sound as far as it goes, but there are serious omissions: no mention of the order in which casualties should be evacuated; no suggestions of priority for abdominal wounds; no stress on the importance of avoiding exposure while applying dressings—the illustrations suggest the casualty should first be stripped naked; no mention of the simple expedient of up-ending the stretcher to clear the bronchi of blood or other fluid, or of the danger of forceful artificial respiration in chest injuries; no suggestion that all gassed cases should be transported lying flat. The best section describes the order for dealing with casualties. The appeal of this book would be wider were it produced as a "Penguin" for ninepence, than for four shillings in its present form.

**Common Skin Diseases.** Seventh Edition. By A. C. ROXBURGH, M.D., F.R.C.P. Pp. xxxii., 454. Illustrated. London: H. K. Lewis & Co. Ltd. 1944. Price 18s. net.—The latest edition of this well-known text-book on dermatology has been considerably expanded and brought up to date, without any appreciable increase in bulk, so that the volume remains of convenient size. The paper and print are good, and the many illustrations, which include eight coloured plates, are excellent. The chapter dealing with scabies describes fully the latest methods of treatment of this infestation, with special reference to the findings of wartime research workers, such as Mellanby. There is valuable information on the subject of industrial dermatitis, its causation and prophylaxis. A whole chapter is devoted to a summary of the recent work on avitaminosis, and the skin lesions associated with vitamin deficiencies are described in detail. A final section deals with the condition known as immersion foot, and its treatment; also Dr. Roxburgh's own experiences in the treatment of such chronic infections as blepharitis and sycosis barbae with penicillin ointment. Several of the rarer dermatoses are mentioned, which renders the book of value to senior practitioners as well as to students and clinical assistants, for whom it was originally intended.

**Massage and Remedial Exercises.** Sixth Edition. By NOEL M. TIDY. Pp. viii., 480. Illustrated. Bristol: John Wright & Sons Ltd. 1944. Price 25s. net.—This encyclopædic *vade-mecum* for physiotherapists has now gone through six editions in twelve years, two of these during the war. The indication is that there is a good demand for the book. The chapters on fractures have been enlarged and modernized (with the help of Watson-Jones's *Fractures*), otherwise there appear to be only minor changes and additions. We have little to add to our critical appreciations of the previous editions, except that this time many of the illustrations are very badly printed. However, this may be the result of bomb damage, which the publishers have suffered on at least two occasions.

**Preparation and After-care of Surgical Patients.** By HUGH C. ILGENFRITZ, M.D., RAWLEY M. PENICK, JUNR., Ph.B., M.D., F.A.C.S., and URBAN MAES, M.D., D.Sc., F.A.C.S. Pp. 532. Illustrated. London: Henry Kimpton. 1942. Price 25s. net.—This is a most excellent handbook, and a sound and up-to-date guide to surgeon and house-surgeon alike. Two principles are repeatedly emphasized. First, that complications are much easier to prevent than they are to treat: secondly, that post-operative care begins in the operating room. The physiological basis for each therapeutic measure advocated is discussed, and it is demonstrated that much of the future progress in surgery will develop from closer co-operation between scientific investigator and practising surgeon. There is a certain amount of repetition from one chapter to another, but the whole is clearly arranged and indexed for ready reference.

**Physical Signs in Clinical Surgery.** Ninth Edition. By HAMILTON BAILEY, F.R.C.S. Pp. viii., 350. Illustrated. Bristol: John Wright & Sons Ltd. 1944. Price 25s. net.—The ninth edition of this popular book is a triumph over adversity, both for the author, who lost nearly all his illustration blocks, and for the publishers, whose premises were twice destroyed by enemy air-raids. Nevertheless, it is as well turned out as ever, and as we expect in a book by Hamilton Bailey, the pictures average two on every page, and by themselves, even without the text, provide a liberal education. Methods of correct examination are shown, as well as almost every variety of surgical ailment or injury that lends itself to pictorial representation. The book is primary designed for students in the fourth to sixth year of their studies, but the doctor who glances through the pictures for a few minutes every now and then when he has a few minutes to spare will almost certainly be helped sooner or later to a correct diagnosis he might otherwise have missed. The text, if not so likely to catch the eye, is as good as the illustrations. Altogether this is a book of more than ordinary value.

**Amputations and Artificial Limbs.** By R. D. LANGDALE-KELHAM, M.R.C.S., L.R.C.P., and GEORGE PERKINS, M.C., F.R.C.S. Pp. 96. Illustrated. London: Oxford University Press. 1942. Price 5s.—The new Oxford War Manual on Amputations and Artificial Limbs has been written by two acknowledged experts on the subject. All useless matter has been ruthlessly cut out and only the bare essentials retained. One of the best features of the book is its description of how to pass out a limb when it has been fitted, and the points in the fitting on which to approve or disapprove. This excellent little book should be read by all surgeons and doctors who have to deal with amputations, and also by students.

**Handbook of Midwifery.** By COMYNS BERKELEY, M.C., M.D., F.R.C.P., F.R.C.S., M.M.S.A., F.R.C.O.G. Eleventh Edition. Pp. x., 622. Illustrated. London: Cassell & Co. Ltd. 1941. Price 8s. 6d. —That a book should reach eleven editions is, in itself, sufficient recommendation of its excellence and many succeeding generations of Medical Students and Pupil-Midwives have found it a most useful companion. This new edition is of convenient size, well produced and illustrated, whilst the new index is an important feature. It is so arranged that the best page for a particular subject in the text can be readily found, when required. At the same time easy revision is aided. The section on diet for pregnant women also adds to the book's usefulness, and in an appendix further information on "food" is provided. Transfusion, with whole blood, plasma, serum, and saline or saline-glucose solutions is also considered in this appendix. In the past the book had a very wide appeal. In the future one may prophesy that it will prove wider still.

**Scabies.** By KENNETH MELLANBY, Ph.D. Pp. xi., 81. Illustrated. London: Oxford University Press. 1943. Price 5s.—Every medical man will welcome this handy pocket-sized Manual. Clear-cut, concise and excellently printed, a great deal of profit is to be gained from its 81 pages. There are some well drawn figures illustrating the life history of the acarus. The style makes for easy reading and sustained interest. After dealing with and comparing the different methods of treatment, the author comes to the conclusion, in a most convincing manner, that sulphur ointment and benzyl benzoate are the most efficient, whilst others fail in a very definite proportion of cases. Dr. Mellanby's views on the transmission of scabies are concise. He considers the disease is a domestic one, and corroborates the opinion of the Ministry of Health that disinfestation of clothes is not necessary, but considers bathing with soap, water and a flannel valuable in dirty cases. He states that the ordinary process of laundering should be sufficient to exterminate the parasite. He makes a point of the way in which the mite attacks the sexes differently, and also certain sections of the community in particular, such as soldiers. His differentiations of diagnosis are very explicit. This is a most excellent little book: the author is to be congratulated on his achievement.

